# Evodiamine derivatives improve cognitive abilities in APP^swe^/PS1^ΔE9^ transgenic mouse models of Alzheimer's disease

**DOI:** 10.1002/ame2.12126

**Published:** 2020-06-29

**Authors:** Shuo Pang, Caixian Sun, Shan Gao, Yajun Yang, Xiandao Pan, Lianfeng Zhang

**Affiliations:** ^1^ Key Laboratory of Human Disease Comparative Medicine National Health Commission of China (NHC) Institute of Laboratory Animal Science Peking Union Medical College Chinese Academy of Medical Sciences Beijing China; ^2^ Beijing Engineering Research Center for Experimental Animal Models of Human Diseases Institute of Laboratory Animal Science Peking Union Medical College Chinese Academy of Medical Sciences Beijing China; ^3^ Beijing Key Laboratory of Active Substance Discovery and Drug ability Evaluation Institute of Material Medical Chinese Academy of Medical Sciences and Peking Union Medical College Beijing China; ^4^ Neuroscience Center Chinese Academy of Medical Sciences Beijing China

**Keywords:** evodiamine derivatives, mouse model, neuroprotective, spatial memory

## Abstract

**Background:**

Alzheimer's disease (AD) is a complex neurodegenerative disease. Due to the complexity of its molecular pathogenesis and the interaction of the numerous factors involved, the etiology and pathogenesis of AD have not been fully elucidated. Therefore, effective treatment for AD remains to be developed. Evodiamine, a quinolone alkaloid, has been found to improve learning and memory ability to in the APP^swe^/PS1^△E9^ mouse model of dementia. However, the cytotoxicity and physicochemical properties of evodiamine have limited its use in the treatment of AD.

**Methods:**

Evodiamine and its derivatives were effectively synthesized by EDCI‐mediated condensation at room temperature. These target compounds contained 1 thio‐ and 21 oxo‐evodiamine derivatives with different substituted groups. The cytotoxicity of evodiamine and its derivatives and the neuroprotective effects of the evodiamine derivatives against H_2_O_2_‐induced cell loss in SH‐SY5Y cells were investigated using the WST‐8 assay. The Morris water‐maze test was used to detect the effect of evodiamine and its derivatives on improving learning and memory in APP^swe^/PS1^△E9^ mice.

**Results:**

In this study, a series of oxo‐ and thio‐evodiamine derivatives was synthesized. Several derivatives showed lower cytotoxicity and stronger neuroprotective effects than evodiamine and elicited enhanced cognitive improvement, especially in the test of spatial memory in APP^swe^/PS1^△E9^ mice.

**Conclusion:**

Our study provides insights for developing novel evodiamine derivatives for chemical intervention and treatment of AD.

## 1 INTRODUCTION

Alzheimer's disease (AD) is a complex neurodegenerative disease that affects more than 35 million people worldwide. Memory loss and cognitive impairment can cause death 3‐9 years after diagnosis.^1^ The main pathological features of AD are senile plaques formed by amyloid deposition and tangles formed by tau protein hyperphosphorylation.^2^ AD is mainly divided into genetic and non‐genetic forms. Patients with genetic AD inherit a familial autosomal dominant form of the disease, presenting as early‐onset dementia, usually occurring between the ages of 30 and 60. This familial form of AD is caused by mutations in genes such as *APP*, *PSEN1* and *PSEN2*.^3^ These gene mutations lead to the accumulation of amyloid beta protein outside neurons by affecting amyloid beta production, the Aβ‐42/Aβ‐40 ratio, or the structure of amyloid beta.^2^ More than 99% of AD is non‐genetic, and sufferers tend to develop the disease later in life, usually after the age of 65 years. Because of the complexity of its molecular pathogenesis and the interaction of the many factors involved in the disease, the etiology and developmental mechanisms of AD have not yet been fully elucidated. Therefore, an effective treatment for AD remains to be developed.^4^

APP^swe^/PS1^ΔE9^ double transgenic mice express two major mutations in the human APP gene, as well as human PS1 mutations knocked‐in into the mouse PS1 gene. These mice were bred on a C57BL/6J background.^5^ The APP^swe^ transgene encodes a mouse–human hybrid with the mouse sequence in the extracellular and intracellular regions and a human sequence within the Aβ domain with Swedish mutations K594N/M595L. The PS1^ΔE9^ transgene encodes the Δexon9 human presenilin‐1. This mouse shows spatial memory deficits at 3 months of age and senile plaques in brain tissue at 4.5 months of age.^6^ Therefore APP^swe^/PS1^ΔE9^ double transgenic mice develop behavioral phenotypic and pathological features which make them useful as an AD model.

Chinese herbal medicine, an important element of Chinese medicine, is becoming increasingly popular among physicians and patients.^7^, ^8^ Many medical plant preparations are marketed to the public for various ailments, including Alzheimer's disease.^9^ Evodiamine is a quinazolinone alkaloid isolated from the fruit of fructus evodia, which has been used to treat cancer, cardiovascular disease, headaches, abdominal pain, postpartum bleeding, dysentery and amenorrhea.^10^, ^11^, ^12^, ^13^ In addition, some studies have found that evodiamine can reduce overexpression of cytokines IL‐1β, IL‐6, TNF‐α, inhibit the inflammatory response and overactivation of glial cells in the brain, and improve the learning and memory ability of APP^swe^/PS1^ΔE9^ dementia model mice.^6^

However, the cytotoxicity of evodiamine is relatively high and its physicochemical properties are not suitable for pharmacological use.^14^ These disadvantages limited its therapeutic use. In order to discover novel and potent lead compounds for AD treatment, we developed an efficient method for generating quinazolinone alkaloids via carbodiimide‐mediated condensation between carbolines with anthranilic acids.^15^ This provided potential for increasing the scaffold diversity of evodiamine. In this study, a series of oxo‐ and thio‐evodiamine derivatives was designed and synthesized using this approach and their potential roles for treatment of AD were investigated.

## 2 MATERIAL AND METHODS

### 2.1 Synthesis

Substituted o‐hydroxybenzoic acid (1.2 mmol), 4,9‐dihydro‐3*H*‐pyrido[3,4‐b] indole (1.0 mmol), 1‐(3‐dimethylaminopropyl)‐3‐ethylcarbodiimide hydrochloride (EDCI, 1.4 mmol), and CH_2_Cl_2_ (3.0 mL) were combined and the reaction mixture was stirred at room temperature. After 4 hours, the reaction mixture was diluted with 30 mL of CH_2_Cl_2_ and then washed with saturated aqueous NaHCO_3_ (20 mL), H_2_O (20 mL), and brine (20 mL). The organic phase was dried over Na_2_SO_4_, filtered, and concentrated under reduced pressure. The residue was purified by column chromatography on silica gel (CH_2_Cl_2_: MeOH = 100:1) to yield the target compounds. The structure of the target compounds was determined by ^1^H NMR and HRMS.

### 2.2 Cell culture

Human neuroblastoma cells (SH‐SY5Y) and human hepatocellular carcinoma (HepG2) cells were cultured in Dulbecco's modified Eagle's medium (DMEM), supplemented with 10% fetal bovine serum (FBS), 2 mM l‐glutamine and 0.1 mg/mL antibiotic penicillin streptomycin solution. All cells were cultured in a humidified atmosphere of 5% CO_2_ at 37°C.

### 2.3 Animals

APP^swe^/PS1^ΔE9^ double‐transgenic mice were maintained on a C57BL/6J genetic background. The mice developed spatial memory deficits at 3 months of age and senile plaques in brain tissue at 4.5 months of age, as previously reported.^16^ All mice were bred in an AAALAC‐accredited facility, and the procedures were approved by the Animal Care and Use Committee at the Institute of Laboratory Animal Science, Peking Union Medical College (ILAS‐GC‐2015‐002).

### 2.4 Groups and treatment

Four‐month‐old APP^swe^/PS1^ΔE9^ double‐transgenic mice and non‐transgenic littermates were randomly assigned to seven treatment groups identified as: non‐transgenic (NTG, n = 7), APP^swe^/PS1^ΔE9^ double‐transgenic (n = 7), evodiamine (n = 7), evodiamine derivative 49 (n = 7), evodiamine derivative 51 (n = 7), evodiamine derivative 58 (n = 7) and evodiamine derivative 60 treatment (n = 7). Evodiamine and its derivatives were diluted in 20% PEG/PBS solution. In the evodiamine and evodiamine derivatives treatment groups, APP^swe^/PS1^ΔE9^ double‐transgenic mice were administered evodiamine and derivatives 49, 51, 58 and 60 at a dose of 1.6 mg/kg via intraperitoneal injection 5 times a week for 4 weeks. The APP^swe^/PS1^ΔE9^ double‐transgenic and NTG groups were treated with 20% PEG/PBS solution as the placebo control and wild‐type normal control, respectively.

### 2.5 Cell viability assays

Cell proliferation was measured by the WST‐8 assay (Cell Counting Kit‐8, Dojindo). Cells were seeded in 96‐well plates in sextuplicate at a density of 10^4^ cells/well with 100 μL culture medium and cultured for 24 hours. The cells were then treated for 24 hours with evodiamine and its derivatives at a dose of 5 μg/mL. At specific time points 10 μL of CCK‐8 solution was added to the cells which were then incubated for 1 hour at 37°C. The absorbance reading was then measured at 450 nm, and the relative cell viability was calculated by normalizing to the vehicle control.

To determine the neuroprotective ability of the treatment compounds, the SH‐SY5Y cells were seeded in 96‐well plates (10^4^ cells/well) and cultured for 24 hours. The cells were then cultured for 3 hours with H_2_O_2_ added to the wells to final concentration of 150 μmol/L. Evodiamine and its derivatives were then added to the wells at doses of 0.5, 0.05 and 0.005 μg/mL and the cells were cultured for 24 hours. Finally, cell viability was then evaluated by CCK8 assay.

### 2.6 Morris water‐maze test

The protocol of the Morris water‐maze test was modified from the reported methods.^17^, ^18^ Briefly, the water maze is a 100 cm diameter pool with a 15 cm diameter escape platform placed 0.5 cm below the water surface. Since C57BL/6J mice are black, we poured an appropriate amount of white paint into the water to make the water white and opaque, and maintained the water temperature at 22‐24°C. The mice were placed in the water near the side of the pool. East, west, south, and north positions were randomly selected and each mouse was tested once. We recorded the times taken by the animals to find the underwater platform. In previous training sessions, if this time exceeded 60 seconds, we guided the animal to the platform and let the animal stay on the platform for 10 seconds. The mice were then removed and dried with a towel and placed in a cage. Each mouse was trained 4 times a day, with a 2 hour interval between two training sessions, for 5 consecutive days.

The platform was removed on the sixth day and exploration training lasted for 60 seconds. The animals were placed in the water at the side opposite the original platform quadrant. The duration that animals spent in the quadrant where the platform was originally placed and the number of times they entered the quadrant were recorded, and were used as detection indicators of spatial memory. A video tracking system (Noldus Ltd, Ethovision XT, Holland) was used for detection and analysis. The results were expressed as SD ± SEM.

### 2.7 Statistical analysis

The data were analysed by two‐tailed unpaired t‐tests and one‐way ANOVA followed by Tukey's post hoc analysis. Data shown are means ± S.D, with *P* < .05 considered statistically significant.

## 3 RESULTS

### 3.1 Synthesis of evodiamine and its derivatives

**Figure 1:**
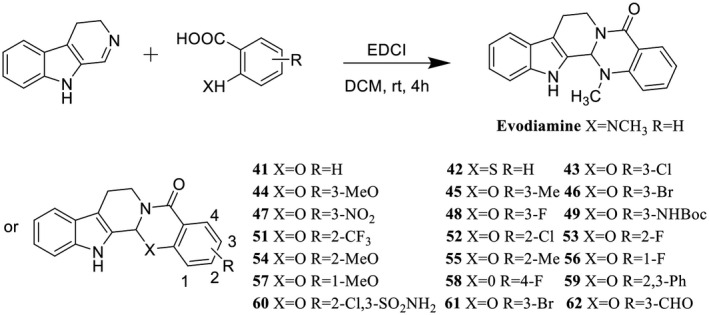
The structure and synthesis of evodiamine and its derivatives. Evodiamine and its derivatives were effectively synthesized by EDCI‐mediated condensation at room temperature

As shown in Figure 1, evodiamine and its derivatives were effectively synthesized by EDCI‐mediated condensation at room temperature. These target compounds contained 1 thio‐ and 21 oxo‐evodiamine derivatives with different substituted groups.

### 3.2 The cytotoxicity of the evodiamine derivatives

**Figure 2:**
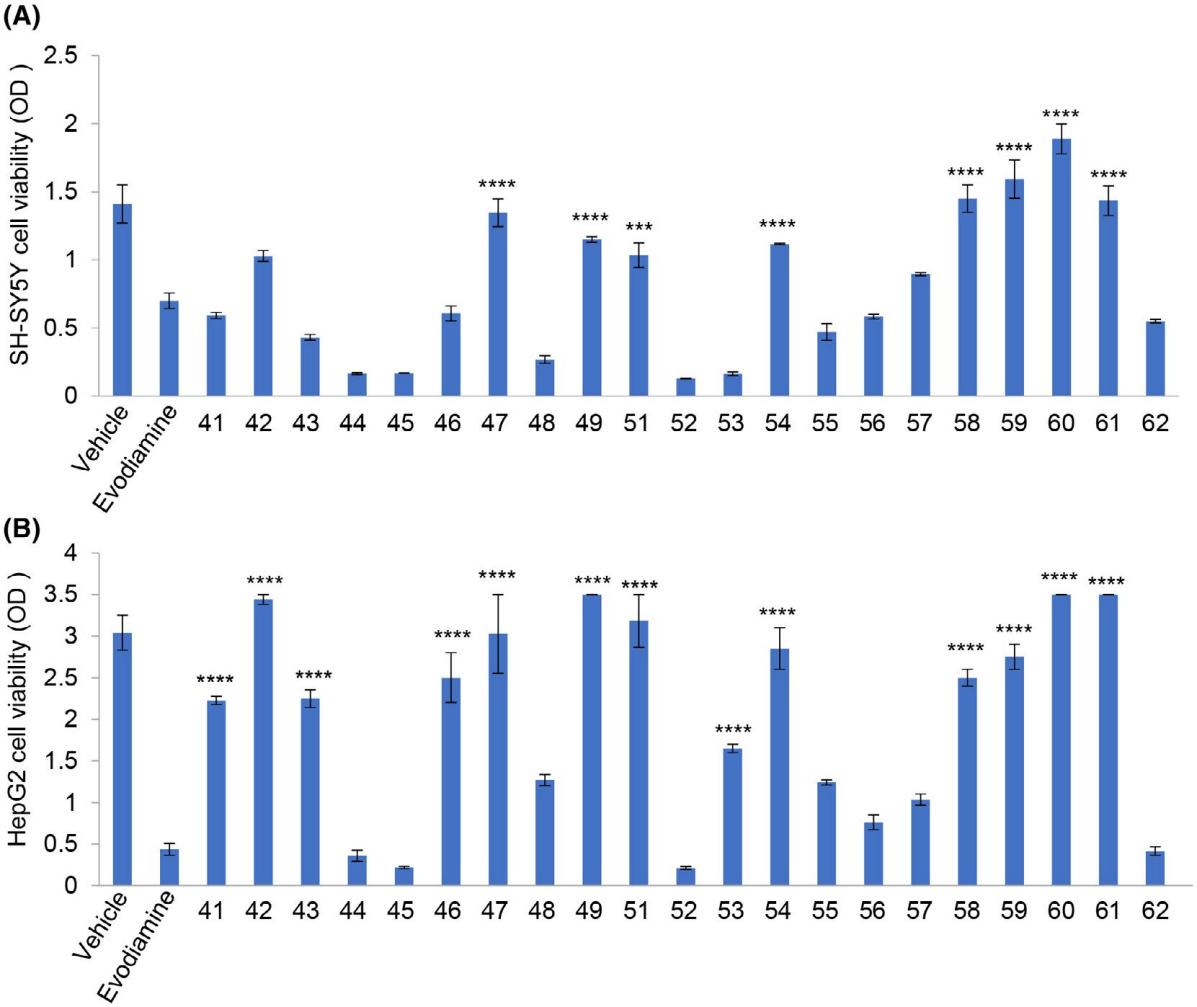
The SH‐SY5Y and HepG2 cells viability was assessed by the CCK‐8 assay. A, Effect of 5 μg/mL evodiamine and its derivatives on SH‐SY5Y cell viability (n = 6, ****P < .0001, ***P < .001, evodiamine derivatives treatment groups vs evodiamine treatment group). B, Effect of 5 μg/mL evodiamine and its derivatives on HepG2 cell viability (n = 6, ****P < .0001, ***P < .001, evodiamine derivatives treatment groups vs evodiamine treatment group)

The cytotoxicity of evodiamine and its derivatives (41‐49 and 51‐62) was first investigated. Compared with the vehicle group, the cell viability of SH‐SY5Y cells treated with evodiamine decreased about 50%. However, the cell viability of SH‐SY5Y cells treated with evodiamine decreased about 50%. However, the cell viability of SH‐SY5Y cells treated with the evodiamine derivatives (47, 49, 51, 54, 58, 59, 60 and 61) increased 91.7%, 68.8%, 52.1%, 58.3%, 109.4%, 131.3%, 170.8%, 110.4% (Figure 2A, n = 6, *P* < .001), respectively. A similar increase in cell viability was also observed in HepG2 cells (Figure 2B, n = 6, *P* < .001). These results indicated that the cytotoxicity of evodiamine derivatives 47, 49, 51, 54, 58, 59, 60 and 61 was obviously decreased compared to evodiamine in both of SH‐SY5Y and HepG2 cells.

### 3.3 The neuroprotective effects of the evodiamine derivatives against H2O2‐induced cell loss in SH‐SY5Y cells

**Figure 3:**
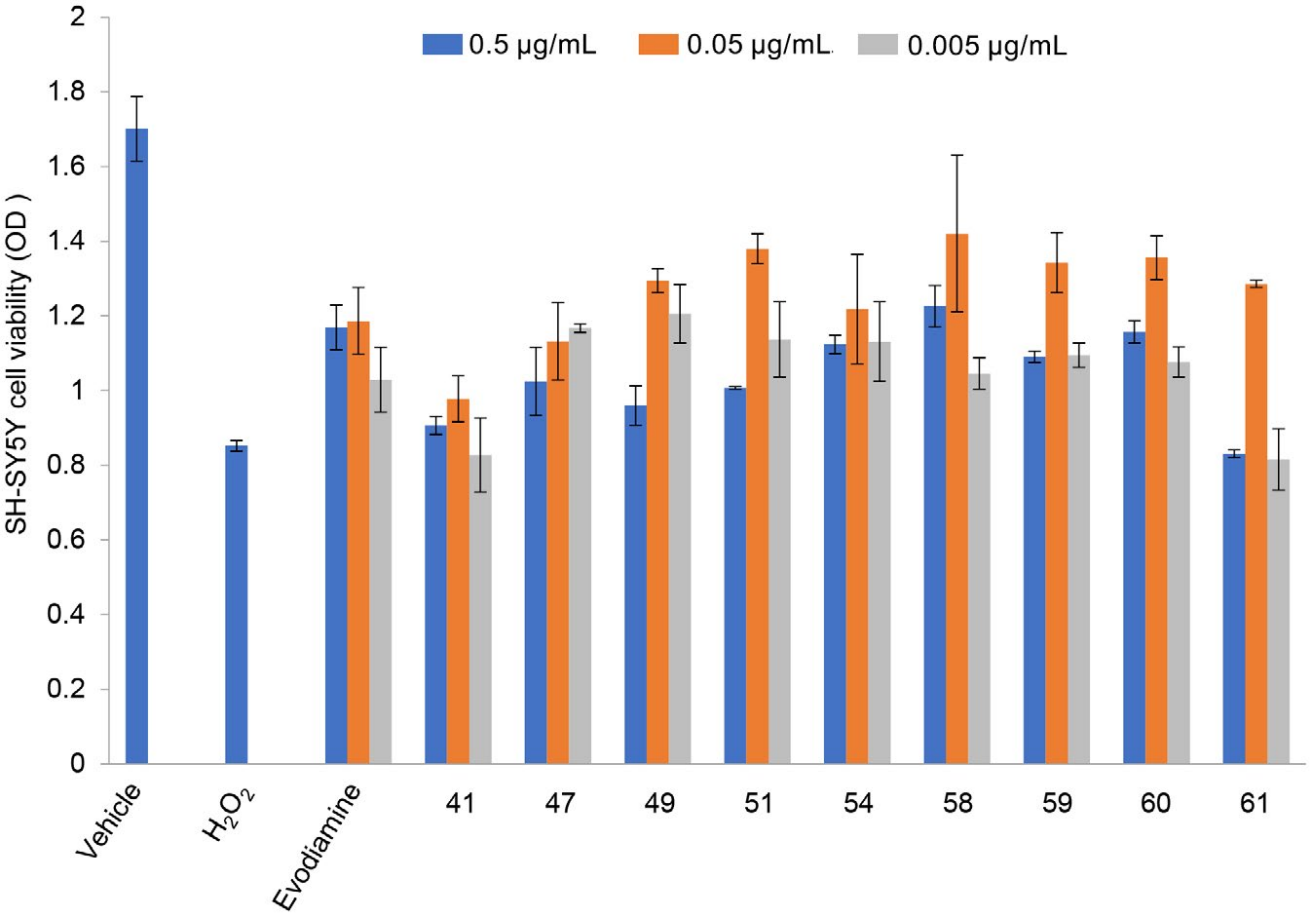
Effects of evodiamine and its derivatives on H_2_O_2_‐induced cytotoxicity in SH‐SY5Y cells determined by CCK‐8 assay. The SH‐SY5Y cells were seeded in 96‐well plates (10 000 cells/well) and cultured for 24 h. Evodiamine and its derivatives (41, 47, 49, 51, 54, 58, 59, 60 and 61) were then added to the wells at doses of 0.5 µg/mL, 0.05 µg/mL and 0.005 µg/mL and the cells were cultured for 3 h. H_2_O_2_ was then added to the wells at a final concentration of 150 μmol/L and the cells were cultured for 24 h. Cell viability was then evaluated by CCK8 assay. The values shown are means ± SE (n = 3)

Based on the results of the cell cytotoxicity experiments, evodiamine derivatives 41, 47, 49, 51, 54, 58, 59, 60 and 61 were selected to further evaluate their neuroprotective effects. Compared with the vehicle group, the cell viability of SH‐SY5Y cells treated with 150 μM H_2_O_2_ decreased by about 50%. Evodiamine and its derivatives (49, 51, 54, 58, 59 and 60) significantly improved the viability of the H_2_O_2_‐treated SH‐SY5Y cells (Figure 3, n = 3), indicating a significant neuroprotective effect. However, there was no significant difference in cell viability between the treatments with evodiamine and its derivatives.

### 3.4 Treatment with evodiamine derivatives increased spatial learning and memory in APP^swe^/PS1^ΔE9^ transgenic mice

**Figure 4:**
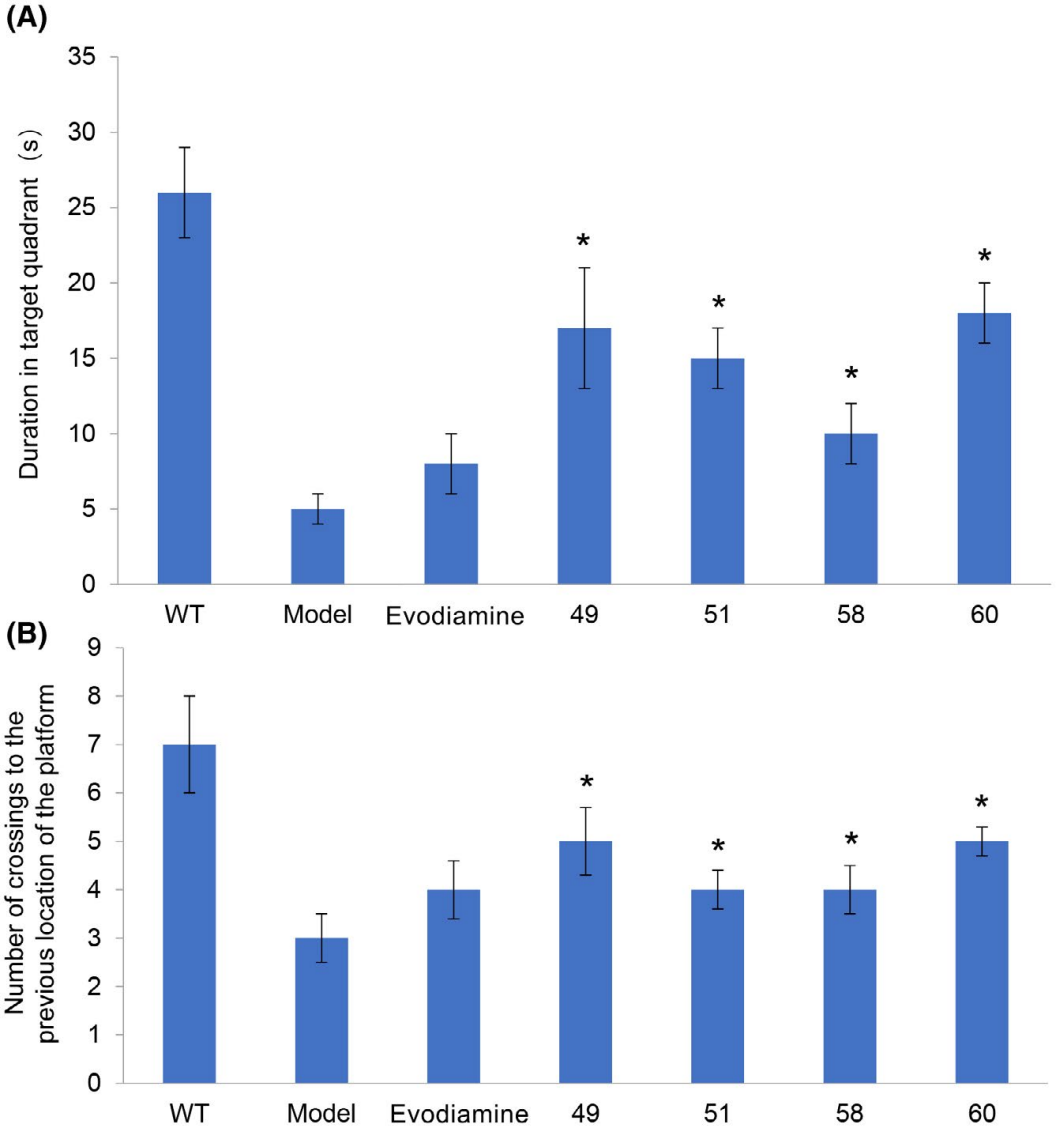
Behavioral performance of animals in the Morris water maze. A, Time spent in the quadrant that previously contained the platform (n = 7, *P < .05, evodiamine derivatives 49, 51, 58 and 60 vs APP^swe^/PS1^ΔE9^). B, Number of crossings to the previous location of the platform (n = 7, *P < .05, evodiamine derivatives 49, 51, 58 and 60 vs APP^swe^/PS1^ΔE9^)

We used APP^swe^/PS1^ΔE9^ transgenic mice to explore the effect of evodiamine and its derivatives on learning and memory, using the Morris water‐maze test. After 4 weeks of treatment, the residence durations in the target quadrant of the mice treated with evodiamine and its derivatives (49, 51, 58 and 60) was significantly higher than that of the untreated APP^swe^/PS1^ΔE9^ transgenic mice group (Figure 4A,B, n = 7, *P* < .001), and the effect of the evodiamine derivatives (49, 51, 58 and 60) was stronger than that of evodiamine. Compared with the APP^swe^/PS1^ΔE9^ group, the number of times that mice crossed the target quadrant increased significantly in the evodiamine and derivatives groups. Among the derivatives groups, the improvements recorded for groups 49 and 60 were more obvious than that of the evodiamine group.

## 4 DISCUSSION

Scaffold diversity has proved to be a powerful strategy for the discovery of novel lead compounds. In this study, a series of oxo‐ and thio‐evodiamine derivatives was synthesized using our reported protocol. In contrast to simple derivatization, our protocol provided an effective route for enhancing the scaffold of evodiamine to increase chemical diversity (Figure 1).

Increasingly in vitro cell models are used to improve the translation value of in vivo models and provide a deeper understanding of the pathological mechanisms of diseases.^19^ The main characteristic of AD is the consumption of acetylcholine, which causes the degeneration of cholinergic neurons in the hippocampus.^20^ Neuroblastoma cells come in both undifferentiated and differentiated forms. Immature cholinergic neurons are undifferentiated, while mature cholinergic cells acquire the capcity to differentiate. These similarities to AD make neuroblastoma cells very useful in drug development.^21^, ^22^ Therefore, we selected SH‐SY5Y cells, a neuroblastoma cell line, and HepG2 cells,^23^ which are sensitive to drug toxicity, to analyze the cytotoxicity of evodiamine and its derivatives. Our results indicate that evodiamine and some of its derivatives have high cytotoxicity. However, evodiamine derivatives 49, 51, 54, 58, 59 and 60 have substantially no side effects on neurons and liver cells (Figure 2).

During the brain aging process in female AD patients, mitochondrial respiration decreases and mitochondrial hydrogen peroxide production increases.^24^ Over the past few decades, the neuroprotective activity of many natural compounds has been evaluated for its protective effect on H_2_O_2_‐induced cell damage in SH‐SY5Y and other related neuronal cell lines.^25^, ^26^, ^27^, ^28^ Our results showed that evodiamine and its derivatives 49, 51, 54, 58, 59 and 60 displayed increased neuroprotective effects (Figure 3).

Furthermore, using the Morris water‐maze test, we showed that treatment with evodiamine and its derivatives for 4 weeks can improve spatial learning and memory in APP^swe^/PS1^ΔE9^ transgenic mice with AD symptoms at 5 months of age (Figure 4). Compared with evodiamine, our derivatives numbers 49, 51, 58 and 60 significantly improved the spatial memory ability of APP^swe^/PS1^ΔE9^ transgenic mice.

In this study, a series of oxo‐ and thio‐evodiamine derivatives was designed and synthesized using the strategy of scaffold diversification. Some of the derivatives showed lower cytotoxicity, and stronger neuroprotective effects, and elicited significant improvements in spatial memory in the APP^swe^/PS1^ΔE9^ AD mouse model. This study further explores the use of evodiamine as a treatment for AD, and also provides insights into developing novel and potent evodiamine derivatives for chemical intervention in AD.

## CONFLICT OF INTEREST

None.

## AUTHOR CONTRIBUTIONS

YY, XP and LZ designed the experiment. YY, XP, SP, CS and SG performed the experiment. SP and YY worked on the statistical analysis of the data. YY, XP and LZ prepared the manuscript. All authors read and approved the final manuscript.
